# Scanning electron microscopy evaluation of enamel surfaces using different air-polishing powders in the orthodontic setting: an in vitro study

**DOI:** 10.1007/s00056-023-00466-2

**Published:** 2023-05-05

**Authors:** Philipp Ratzka, Paul Zaslansky, Paul-Georg Jost-Brinkmann

**Affiliations:** 1 Department of Orthodontics and Dentofacial Orthopedics, Charité - Universitätsmedizin Berlin, Corporate Member of Freie Universität Berlin and Humboldt-Universität zu Berlin, Aßmannshauser Str. 4–6, 14197 Berlin, Germany; 2 Department of Operative and Preventive Dentistry, Charité - Universitätsmedizin Berlin, Corporate Member of Freie Universität Berlin and Humboldt-Universität zu Berlin, Aßmannshauser Straße 4-6, 14197 Berlin, Germany

**Keywords:** Enamel roughness, Prophylaxis, AIR-FLOW®, Erythritol, Sodium bicarbonate, Schmelzrauheit, Prophylaxe, AIR-FLOW®, Erythritol, Natriumbikarbonat

## Abstract

**Purpose:**

The aim of this in vitro study was to quantify and compare changes of the enamel surface caused by periodical use of different air-polishing powders during multibracket therapy.

**Methods:**

Bovine high-gloss polished enamel specimens were air-polished using an AIR-FLOW® Master Piezon with maximum powder and water settings. Each specimen was blasted with sodium bicarbonate (AIR-FLOW® Powder Classic, Electro Medical Systems, Munich, Germany) and erythritol (AIR-FLOW® Powder Plus, Electro Medical Systems). Blasting duration was adapted to the powders’ cleaning efficacy and corresponded to 25 air-polishing treatments in a patient with braces. A spindle apparatus ensured uniform guidance at a distance of 4 mm and a 90° angle. Qualitative and quantitative assessments were performed with the use of low vacuum scanning electron microscopy. Following external filtering and image processing, arithmetical square height (S_a_) and root mean square height (S_q_) were determined.

**Results:**

Both prophy powders caused a significant increase in enamel roughness. Surfaces blasted with sodium bicarbonate (S_a_ = 64.35 ± 36.65 nm; S_q_ = 80.14 ± 44.80 nm) showed significantly (*p* < 0.001) higher roughness than samples treated with erythritol (S_a_ = 24.40 ± 7.42 nm; S_q_ = 30.86 ± 9.30 nm). The observed defects in enamel structure caused by sodium bicarbonate extended across prism boundaries. Prism structure remained intact after air-polishing with erythritol.

**Conclusion:**

Both applied air-polishing powders led to surface alterations. Despite shorter treatment times, sodium bicarbonate was significantly more abrasive than erythritol. Clinicians must compromise between saving time and abrasively removing healthy enamel.

**Supplementary Information:**

The online version of this article (10.1007/s00056-023-00466-2) contains supplementary material, which is available to authorized users.

## Abstract (de)

**Ziel:**

Das Ziel dieser In-vitro-Studie war es, Veränderungen der Schmelzoberfläche, die durch die periodische Verwendung verschiedener Air-Polishing-Pulver während der Multibracket-Therapie verursacht wurden, zu quantifizieren und zu vergleichen.

**Methoden:**

Hochglanzpolierte Rinderschmelzproben wurden mit einem AIR-FLOW® Master Piezon mit maximalen Pulver- und Wassereinstellungen bestrahlt. Jede Probe wurde mit AIR-FLOW® Pulver Classic (Natriumbikarbonat) und AIR-FLOW® Pulver Plus (Erythritol) gestrahlt. Die Strahldauer wurde an die Reinigungswirkung der Pulver angepasst und entsprach 25 Pulver-Wasserstrahl-Behandlungen bei einem Patienten mit Brackets. Eine Spindelvorrichtung gewährleistete eine gleichmäßige Führung des Handstücks im Abstand von 4 mm und einem Winkel von 90°. Mithilfe eines Rasterelektronenmikroskops wurden qualitative und quantitative Bewertungen durchgeführt. Nach externer Filterung und Bildbearbeitung wurden die mittlere arithmetische Höhe (S_a_) und die mittlere quadratische Höhe (S_q_) bestimmt.

**Ergebnisse:**

Beide Air-Polishing-Pulver verursachten eine signifikante Zunahme der Schmelzrauheit. Mit bikarbonathaltigem Pulver (S_a_ = 64,35 ± 36,65 nm; S_q_ = 80,14 ± 44,80 nm) bestrahlte Oberflächen zeigten eine signifikant (*p* < 0,001) höhere Rauheit als mit erythritolhaltigem Pulver (S_a_ = 24,40 ± 7,42 nm; S_q_ = 30,86 ± 9,30 nm) behandelte Proben. Die beobachteten Defekte in der Schmelzstruktur, die durch das bikarbonathaltige Pulver verursacht wurden, überschritten die Prismengrenzen. Nach dem Einsatz von erythritolhaltigem Pulver blieb die Prismenstruktur intakt.

**Schlussfolgerung:**

Beide getesteten Air-Polishing-Pulver führten zu Oberflächenveränderungen. Trotz kürzerer Behandlungszeiten zeigte sich das bikarbonathaltige Pulver deutlich abrasiver als das erythritolhaltige. Kliniker müssen einen Kompromiss zwischen Zeitersparnis und abrasivem Entfernen von gesundem Zahnschmelz eingehen.

## Introduction

Initial carious lesions appear opaque on smooth surfaces of teeth due to microporosities in the enamel structure and are, therefore, known as white spot lesions [21]. They are significantly less common than fissure or interproximal caries in the general population. In a large Scottish youth study, smooth surface caries accounted for only 13% of all carious lesions [11]. Reasons for this are reduced plaque retention, sufficient saliva flushing and better cleanability. The use of multibracket appliances leads to an increased incidence of white spot lesions and the esthetically important upper anterior teeth are most frequently affected [14]. While the incidence of white spot lesions is reported to be between 26 and 54% if no prophylaxis program is implemented [1, 7, 10], it can be reduced to 7.4% by critical patient selection and prophylactic measures [33, 41]. For this purpose, topical fluoride, fluoride-releasing adhesives, smooth surface sealants, or products for chemical plaque inhibition are used [16, 34]. Regular professional tooth cleaning in combination with oral hygiene instructions and motivation are also recommended for preventing white spot lesions [22, 40]. Air-polishing devices are ideal for orthodontic patients as wires and auxiliaries can remain in situ during air-polishing [3, 38]. When used in the practice, air-polishing is less time consuming than tooth cleaning with curettes or rubber cups [15, 28]. A recent randomized controlled trial showed that cleaning with an air-polishing device leads to lower plaque scores than rubber cup polishing [39].

There are many prophylactic powders for air-polishing devices with different abrasive qualities [15]. Some earlier references agreed that sodium bicarbonate powder can be used on healthy enamel as standard. However, the application on root surfaces and demineralized enamel led to high surface abrasion and should be avoided [5, 23, 26]. For this indication erythritol-based prophylactic powders appear advantageous, as they are considered to be all-rounders due to their lower abrasiveness [15]. However, limited quantitative data have been published about the abrasiveness of erythritol powders. Glycine powder, which is also widely used, is primarily recommended for cleaning root surfaces [6]. Abrasive powders may induce surface roughness changes, which were shown to depend on the chosen prophylactic device and its settings [19, 20].

In previous studies about powder abrasiveness, test specimens were mostly exposed to long-term blasting corresponding to years of periodical prophylaxis [3, 9, 19, 20, 36]. In a preceding study, we showed that those blasting durations are too long to represent periodical air-polishing during multibracket therapy and we also proved that blasting durations differ depending on prophy powder choice [30]. To our knowledge, there is no study yet, which considers powder-specific blasting durations when examining surface alterations after air-polishing. In order to quantify surface alterations, unevenness in the surface height, known as surface roughness, can be measured. Nowadays optical roughness measurement systems are most common, but many are not ideal for reflective and translucent surfaces, such as enamel. Therefore, we adapted a scanning electron microscopy-based (SEM) approach, which uses backscattered electrons for shape-from-shading, for roughness measurements in this study.

The purpose of the work presented here was to compare enamel surfaces qualitatively and quantitatively after the use of different prophylactic powders in an orthodontic setting. For better clinical transferability the blasting durations were adapted according to their respective cleaning efficacy.

## Materials and methods

In this in vitro study, bovine incisor enamel surfaces were analyzed using a Phenom XL low-vacuum electron microscope (Thermo Fisher Scientific, Hagen, Germany) to compare the surface micro- and nanotopography after air-polishing using sodium bicarbonate (AIR-FLOW® Powder Classic, Electro Medical Systems, Munich, Germany) and erythritol (AIR-FLOW® Powder Plus, Electro Medical Systems, Munich, Germany) powder.

### Specimen preparation and blasting process

Crowns of permanent bovine incisors were partly embedded in epoxy resin (PUR11, ebalta, Rothenburg o. d. Tauber, Germany) after removal of soft tissue and were stored in a 0.1% thymol solution. The test specimens were ground until a flat area of at least 6 mm × 7 mm in size was created followed by polishing to high gloss using a disc grinder (dia-plus, Walter Messner, Oststeinbek, Germany) and SiC paper (1200 grit, 2400 grit, 4000 grit for 3 min each) loaded with 200 g at 75 rpm under continuous water cooling.

**Fig. 1:**
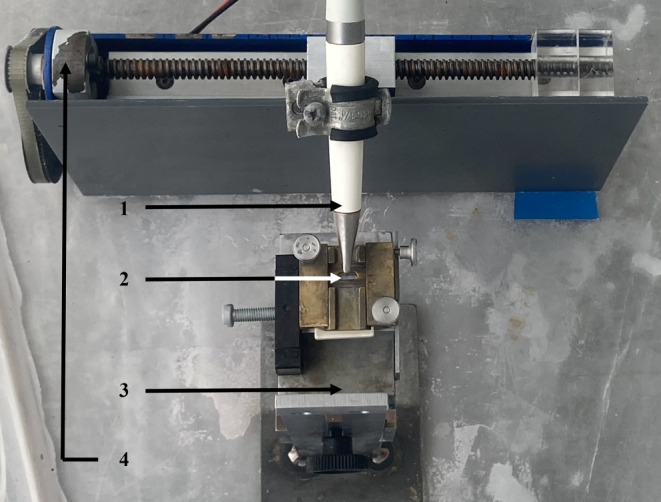
Experimental setup. *1* Handpiece, *2* Slit aperture with sample, *3* Height-adjustable object table with vise, *4* Spindle apparatus Versuchsaufbau. *1* Handstück, *2* Spaltöffnung mit Probe, *3* höhenverstellbarer Objekttisch mit Schraubstock, *4* Spindelapparat

A metal specimen holder with a slit (2 mm × 7 mm, Fig. 1) ensured that the enamel surface was only blasted in the exposed area. This guaranteed that each tooth could be treated with both sodium bicarbonate and erythritol in separate sections. Air-polishing (AIR-FLOW® Master Piezon, Electro Medical Systems, Munich, Germany) was performed on maximum powder and water settings. The handpiece was guided perpendicular to the exposed enamel surface with a spindle apparatus, which also ensured a constant blasting distance of 4 mm as shown in Fig. 1. According to the results of our preceding study [30], a blasting duration of 47.5 s for sodium bicarbonate and 58.5 s for erythritol was chosen. This corresponds to 25 air-polishing treatments in a multibracket patient. The blasting duration was varied by adjusting the spindle motion speed. The powder chambers were refilled to maximum after each test specimen.

After air-polishing, any contamination of the test specimens was eliminated using ethanol followed by compressed air. Before the roughness measurement, the specimens were dried for at least 72 h at 36 °C in a dust-proof container.

### Qualitative and quantitative evaluation

**Fig. 2:**
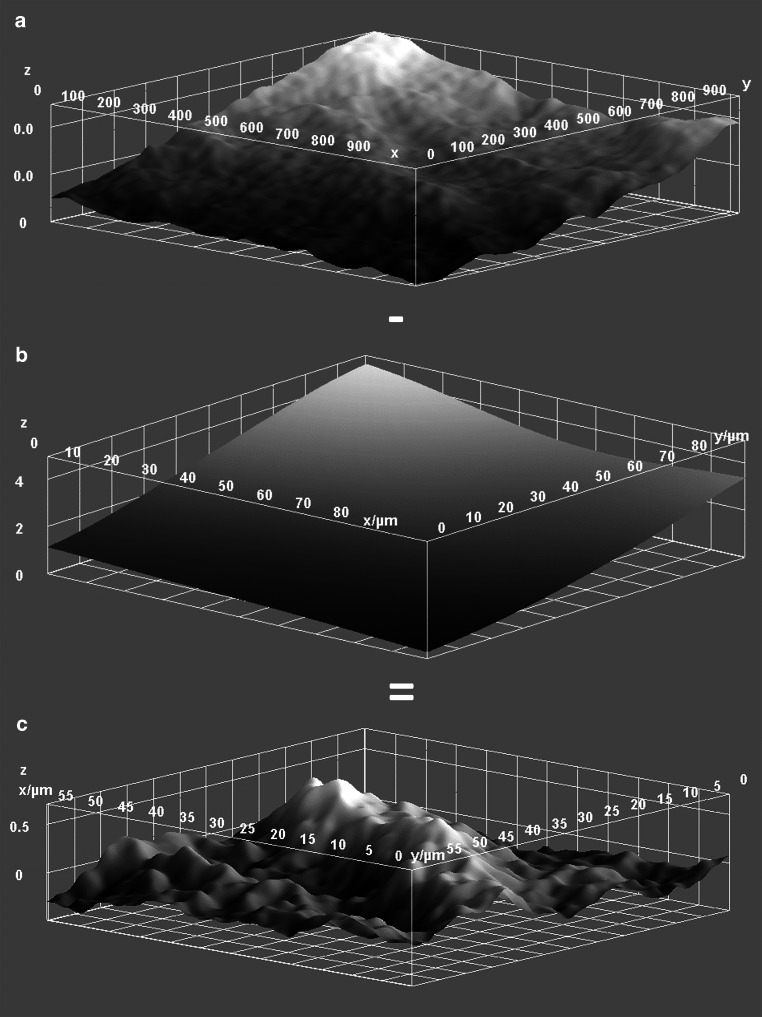
Processing and filtering in Fiji (imagej.net). **a** Three-dimensional surface plot of the primary profile. **b** Surface plot after scaling of the image and application of the Gaussian filter to obtain the wave profile (field of view 100 µm × 100 µm). **c** By subtracting the wave profile from the primary profile, the roughness profile is created. The edges have been cut off because of filtering artifacts, which results in an evaluable field of view of 60 µm × 60 µm Bearbeitung und Filterung in Fiji (imagej.net). **a** Dreidimensionaler Oberflächenplot des Primärprofils. **b** Oberflächenplot nach Skalierung des Bildes und Anwendung des Gauß-Filters zur Gewinnung des Wellenprofils (Gesichtsfeld 100 µm × 100 µm). **c** Durch Subtraktion des Wellenprofils vom Primärprofil entsteht das Rauheitsprofil. Die Kanten wurden aufgrund von Filterungsartefakten abgeschnitten, was zu einem auswertbaren Sichtfeld von 60 µm × 60 µm führt

The enamel surfaces were examined and scanned with a Phenom XL scanning electron microscope (SEM) in three randomly chosen sections of the high-gloss polished and the treated areas devoid of enamel cracks or other artifacts. The energy and imaging conditions of the SEM are listed in Table 1. The built in 3D Roughness Reconstruction Software was used to create three-dimensional topography maps. As surfaces appear curved and tilted due to the applied vacuum and high magnification, the primary profile had to be filtered despite the planar high-gloss polish (Fig. 2). The reconstructions were, therefore, imported into Fiji (imagej.net) for image processing and filtering using a Gaussian filter. The macro, which was used for the processing, is published as Supplementary Table 1.

**Table 1:** Energy and imaging conditions of the Phenom XL Energie- und Bildgebungsbedingungen des Phenom XL

Magnification	3000 × (field of view approximately 100 µm × 100 µm)
Working distance	5 mm ± 1 mm
Vacuum	Low
Voltage	15 kV
Detector	BSD full
Beam intensity	Image

*BSD* Back-Scatter Detector

When selecting the filter size, several matters need to be considered: First, the wave profile must be completely eliminated while the roughness profile has to be preserved. Second, artifacts in the edge area occur in proportion to the size of the Gaussian filter. Therefore, the edges must be cut off after subtracting the wave profile from the primary profile reducing the evaluable area. Considering this, we examined different filter sizes by comparing the resulting three-dimensional (3D) surface plots and profile plots. A filter with σ = 15 was found to isolate the relevant roughness profile while preserving an acceptable large area of approximately 60 µm × 60 µm after artifact cut-off (Fig. 2).

Afterwards the reconstructions were evaluated in Gwyddion 2.55 (gwyddion.net) in terms of arithmetical mean height (S_a_) and root mean square height (S_q_). The calculated values for S_a_ and S_q_ of the three randomly chosen sections of each area were then averaged, respectively.

### Validation of the measurement method

Since filtering determines which roughness profile is isolated, it has a major impact on the measurement. For validation purposes, precision and trueness were tested to determine the accuracy of three different analysis methods:

- No filtering
- Automatic first-order correction of the 3D Roughness Reconstruction Software
- Filtering and image processing in Fiji

Precision was defined as the percentage ratio between the standard deviation and the mean measured roughness from repeated measurements. It was tested by measuring the roughness of the same spot of an enamel test specimen in different positions, rotations, and working distances for each analysis method. Filtering in Fiji had a high precision of 1.82%, whereas precision was low for no filtering (27.71%) and automatic first-order correction (7.70%) in the 3D Roughness Reconstruction Software. The corresponding data are given in Supplementary Table 2.

The ICH Q2 (R1) defines trueness as “closeness of agreement between the value which is accepted either as a conventional true value or an accepted reference value and the value found” [17]. Since there is no reference for our procedure, a metal roughness normal (KNT 4070/03, Halle Präzisions-Kalibriernormale, Edmissen, Germany), which has a repeating profile every 1.25 mm with a known mean roughness of 73.50 nm, was used to check whether the values measured are reasonable. The difference in mean was lower for first-order correction as compared with filtering using Fiji (Supplementary Table 3), but in further consideration of surface measurements, higher roughness values after first-order correction are related to a strong curvature of the profile and do not correlate with high roughness measured after complete filtering with Fiji as one can comprehend in the figures shown in Supplementary Fig. 1. Based on these results, we chose to use Fiji for filtering and image processing in this study.

### Statistical analysis

The statistical evaluations were carried out in Excel (Microsoft 365, Redmond, WA, USA). The following null hypotheses were tested in two-tailed t‑tests assuming different variances with Bonferroni correction at the significance level *p* < 0.017:

- Air-polishing of high-gloss polished enamel surfaces with sodium bicarbonate does not lead to surface roughness changes.
- Air-polishing of high-gloss polished enamel surfaces with erythritol does not lead to surface roughness changes.
- There is no difference in roughness between enamel surfaces blasted with sodium bicarbonate and erythritol.

## Results

### Quantitative surface evaluation

**Fig. 3:**
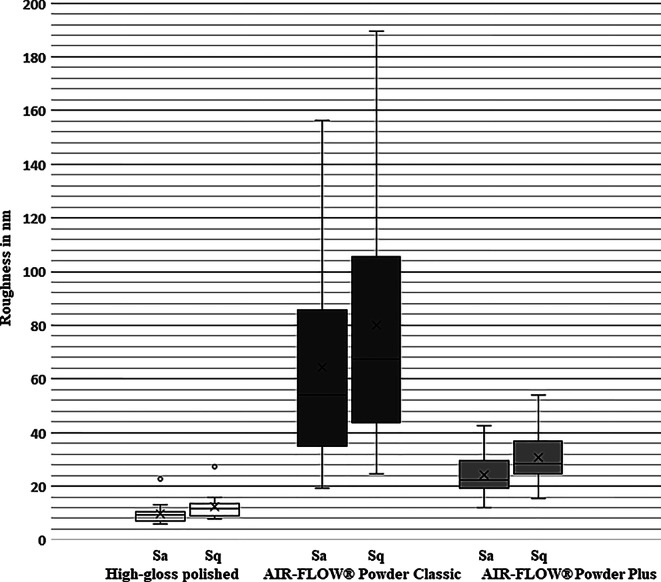
Box and whisker plot illustrating the roughness values (S_a_, S_q_) of high gloss polished surfaces and those treated with sodium bicarbonate (AIR-FLOW® Powder Classic) and erythritol (AIR-FLOW® Powder Plus) Box- und Whisker-Plot zur Darstellung der Rauheitswerte (S_a_, S_q_) von hochglänzend polierten Oberflächen sowie mit Natriumbikarbonat (AIR-FLOW® Powder Classic) und Erythritol (AIR-FLOW® Powder Plus) behandelten Oberflächen

We observed a highly significant increase in surface roughness of polished enamel surfaces (S_a_ of 9.16 ± 4.09 nm; S_q_ of 11.68 ± 4.68 nm) following air-polishing with sodium bicarbonate (S_a_ = 64.35 ± 36.65 nm; S_q_ = 80.14 ± 44.80 nm) and erythritol (S_a_ of 24.40 ± 7.42 nm; S_q_ of 30.86 ± 9.30 nm; *p* < 0.001). Furthermore, enamel surfaces treated with sodium bicarbonate were significantly rougher than surfaces treated with erythritol (*p* < 0.001; Fig. 3). The high deviation of the roughness values in the sodium bicarbonate group stands out, since there were surfaces with values in the range of those which were treated with erythritol and others that showed a S_a_ and S_q_ higher than 150 nm.

### Qualitative surface evaluation

**Fig. 4:**
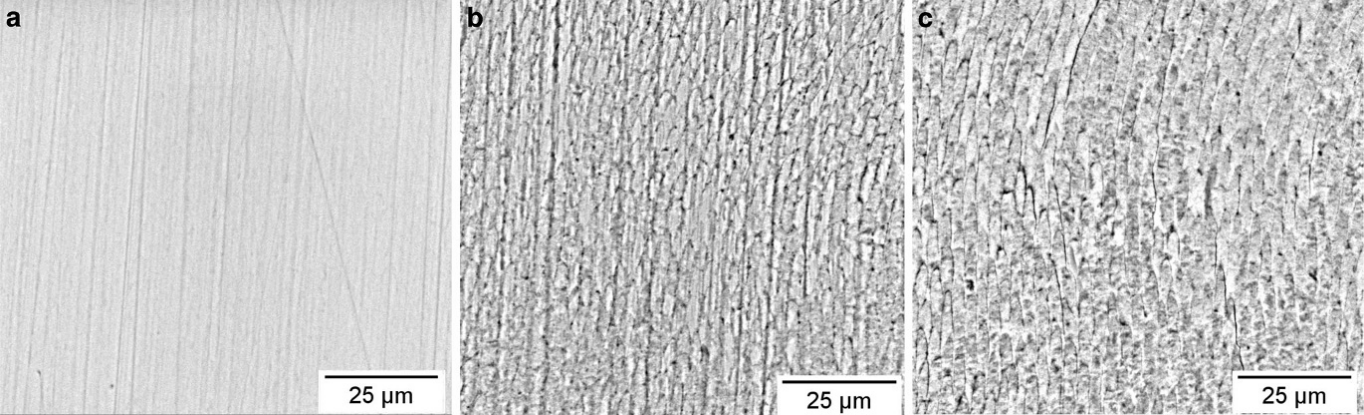
Scanning electron microscope (SEM) pictures (magnification 3000 ×) of high-gloss polished enamel surfaces (**a**) and enamel surfaces treated with erythritol (**b**) and sodium bicarbonate (**c**) Rasterelektronenmikroskopische Aufnahmen (Vergr. 3000 ×) von hochglänzend polierten Schmelzoberflächen (**a**) und von mit Erythritol (**b**) und Natriumbikarbonat (**c**) behandelten Schmelzoberflächen

**Fig. 5:**
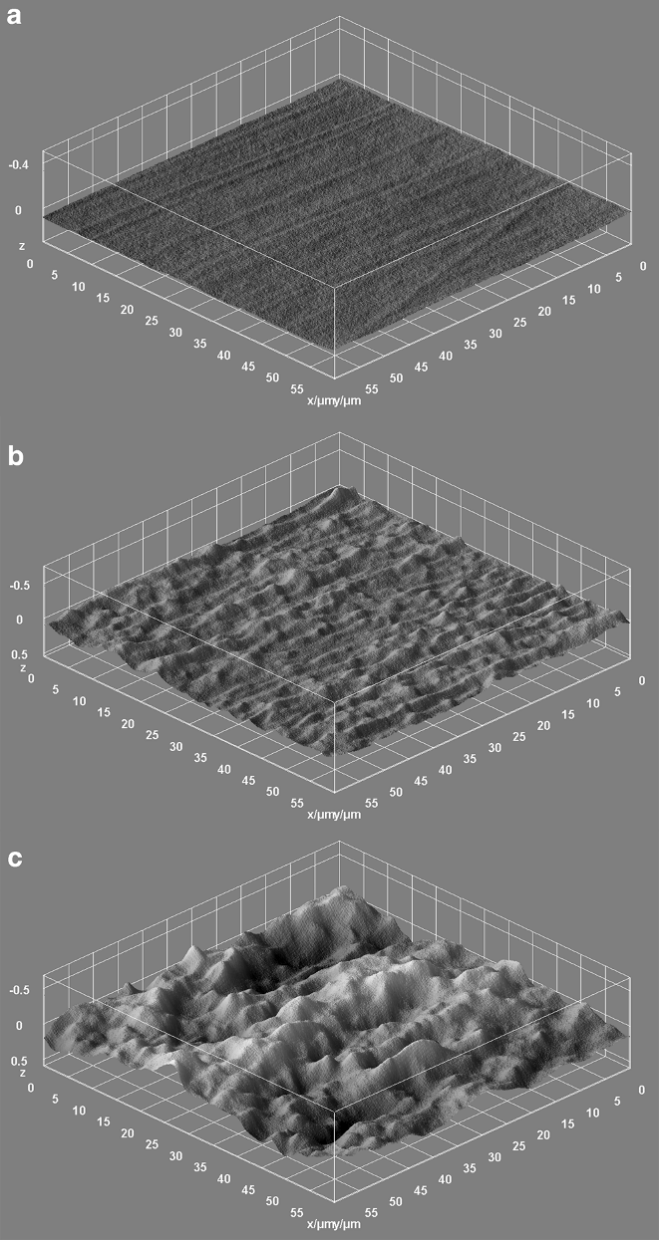
Three-dimensional surface plots of high-gloss polished enamel surfaces (**a**) and enamel surfaces treated with erythritol (**b**) and sodium bicarbonate (**c**) Dreidimensionale Oberflächendarstellung von hochglänzend polierten Schmelzoberflächen (**a**) und von mit Erythritol (**b**) und Natriumbikarbonat (**c**) behandelten Schmelzoberflächen

When examining the polished enamel surfaces with the SEM, delicate polishing marks could be seen. They were still partially visible in most areas blasted with erythritol, while they were no longer detectable in the areas treated with sodium bicarbonate as shown in Fig. 4. Furthermore, enamel prisms were much more exposed after the use of both air-polishing powders compared to the polished areas. One can measure a prism width of approximately 4–5 µm in the SEM images. In the 3D surface plots, they represent as hills of the same size. When comparing the surface plots, it appears that the structure of the prismatic enamel remained largely intact when using erythritol. The abrasion through sodium bicarbonate air-polishing extended beyond the prism boundaries (Fig. 5).

## Discussion

### Quantitative surface evaluation

The present study showed that the surface roughness of polished enamel surfaces (S_a_ = 9.16 ± 4.09 nm; S_q_ = 11.68 ± 4.68 nm) was increased by air-polishing with sodium bicarbonate (S_a_ = 64.35 ± 36.65 nm; S_q_ = 80.14 ± 44.80 nm) and erythritol (S_a_ = 24.4 ± 7.42 nm; S_q_ = 30.86 ± 9.30 nm; *p* < 0.001). According to the results of our procedure validation, it can be assumed that the “true” roughness might be higher than measured here. But “true roughness values” are of theoretical nature and trueness is not critical for intergroup comparisons since it does not affect relative differences. Thus, based on our measurements, it has to be said that sodium bicarbonate leads to rougher surfaces than erythritol (*p* < 0.001).

While the effects of sodium-bicarbonate-based prophy powders on dental enamel have been extensively examined, only few studies investigated the roughness of enamel surfaces after blasting with erythritol powder [32, 36]. Tocha obtained comparable results when using an optical system based on focus variation and examining natural enamel surfaces after stationary air-polishing for 5 s with otherwise identical working parameters (4 mm blasting distance, 90° jet angle, maximum powder and water settings) [36]. He found an increase in S_a_ by 164 nm after air-polishing with sodium bicarbonate and by 26 nm with erythritol. In another study, Sinjari et al. found natural enamel surfaces to be smoothed by blasting with sodium bicarbonate (AIR-FLOW® Powder Classic), while no significant change in roughness was caused by erythritol (AIR-FLOW® Powder Plus) and glycine (AIR-FLOW® Powder Soft) [32]. This astonishing result is probably due to the extremely high natural roughness of the anterior teeth examined (R_q_ = 91.49 µm). In addition, the blasting distance was set at 10 mm, which would lead to an extensive blasting of the soft tissue and the multibracket appliance in clinical practice.

Fratolin et al. [13] and Jost-Brinkmann [19] compared the effect of air-polishing with sodium bicarbonate and different prophylactic pastes on enamel. Fratolin et al. found that air-polishing for approximately 0.25 s/mm^2^ did not lead to higher surface roughness than rubber cup polishing. When blasting duration was increased to 1.5 s/mm^2^, significantly rougher surfaces were observed. In spite of air-polishing for more than 4 s/mm^2^, the results of Jost-Brinkmann showed even smaller or at least not significantly different roughness values and enamel removal for most air-polishing devices and settings compared to rubber cup polishing. According to their results, both studies recommended air-polishing enamel surfaces with sodium bicarbonate in clinical use under certain conditions.

### Qualitative surface evaluation

Camboni and Donnet found intact enamel surfaces after air-polishing with erythritol (AIR-FLOW® Powder Plus), whereas the application of sodium bicarbonate (AIR-FLOW® Powder Classic) changed the surface structure significantly [9]. The current study confirms these results. When analyzing the 3D surface plots, one can clearly see an abrasion beyond prism boundaries after the use of sodium bicarbonate. Furthermore, the polishing marks that still can be seen after air-polishing with erythritol are no longer detectable after sodium bicarbonate usage. Therefore, increased substance removal through sodium bicarbonate must be concluded. This is presumably due to the large particle size of the sodium bicarbonate of approximately 65 µm, whereas the particles of the erythritol are on average 14 µm in size. Since the evaluated field of view was only 60 µm × 60 µm, the smoother surfaces of the erythritol group could be better represented than the more irregular surfaces of the sodium bicarbonate group, which might be the reason for the larger scatter in the roughness values in this group.

### Specimens and blasting process

Bovine enamel specimens are widely used in dental research because they are relatively easy to obtain and their mechanical properties are similar to human enamel [2, 12, 29, 37]. A significant advantage is that they were never exposed to previous dental treatment and are sufficiently massive so that it is possible to prepare large areas of enamel without exposing dentin.

It is known that blasting distance, angle, and duration as well as the powder and water settings of air-polishing devices have an influence on the abrasiveness of air-polishing. Petersilka et al. compared these working parameters and concluded that air-polishing duration had the greatest influence on root dentin removal [25]. In previous studies, the duration was usually set very high corresponding to years of a prophylactic program and differences in cleaning efficiency were not considered [3, 9, 19, 20, 36]. According to recently-determined cleaning performances of the tested prophy powders, we opted to use different blasting times, which correspond to a prophylaxis program during multibracket therapy with 25 air-polishing treatments. This makes it possible to achieve a fair comparison and better transferability of the results of this study into the clinical setting. However, enamel surfaces are exposed to natural abrasion and remineralization processes and consequently a summation of the blasting effects of the individual air-polishing treatments cannot automatically be assumed on the long run [31]. These processes were not considered in the present study, but it can be assumed that the natural abrasion of the buccal enamel has no major effect in the setting of a 2-year orthodontic treatment with monthly professional tooth cleaning. The maximum powder and water settings were selected because this way the cleaning performance is maximized and the influence on the resulting roughness was described as low or as not clearly verifiable for the preceding air-polishing device, AIR-FLOW® S1, on clinically relevant settings [19, 26]. By means of the spindle apparatus, a constant distance of 4 mm and a jet angle of 90° could be ensured. At the same time, stationary blasting was avoided without claiming that the linear spindle movement imitates the complex handpiece guidance during professional tooth cleaning. The main goal was to prevent cratering. As a result and to achieve better control and comparability, the manufacturer’s recommendations for the jet angle were disregarded. However, due to the complex morphology of the vestibular dental surfaces with bonded brackets, these cannot be implemented in vivo either. The powder chamber was filled to maximum after blasting each specimen because the powder release decreases as the fill level lowers [27].

### Measuring method

For surface roughness measurements, mainly tactile methods were used in the past. These devices, however, show a limited resolution due to the size of their stylus tip and can only measure line profiles [8]. Recently, optical methods with higher resolutions are increasingly used. These allow the measurement of surface-related parameters which, in contrast to line parameters, are not orientation-dependent and, therefore, more representative [35]. Several optical measuring systems work with the principle of dynamic focusing, which is disturbed by the translucency of enamel and by the strong reflection of plane polished surfaces. Hence, in the present study, we opted to use electron microscopy where the image is confined to the surface. Specifically, the Phenom XL desktop SEM has integrated software that allows the spatial reconstruction of surface topography using shape-from-shading. This principle is based on different shadows caused by surface reliefs when exposed to electron radiation from different directions. The backscattered electron detector of the Phenom XL consists of four segments with a spatial offset between them. In this way, four images are generated from different directions of the same surface, from which a three-dimensional reconstruction can then be calculated.

Subsequent filtering of the reconstructed primary profile determines which roughness profile is evaluated and thus has a major influence on the results. The reconstruction software has different filtering options. One can manually choose wavelength settings but there are no standards for this application yet. Another possibility is to use the integrated first-order correction for automatic filtering. This leads to a loss of control over the information about which profile is actually filtered. To avoid ambiguity and increase reproducibility, we advanced this method of roughness quantification by exporting the respective heightmaps to Fiji image processing and filtering. Our validation showed that this method had a much higher precision than the automatic first-order correction. Due to the flat high-gloss polish of the enamel samples, the described filtering in the experiment was simplified and could be checked using 3D surface plots. Polishing also granted the advantage that a baseline measurement could be neglected, and repositioning errors were thus ruled out.

### Limitations

Although in vitro studies offer the advantage of great standardization, they are always associated with limited transferability to the clinical situation. However, clinical studies with a higher level of evidence are difficult to carry out on the presented subject because the measurements cannot be done in vivo. Also, erupted teeth are—if indicated—usually extracted at the beginning and not the end of orthodontic therapy. Further limitations exist because the biofilm that must be removed initially protects the enamel surface. In addition, the plane polished enamel surfaces of the specimens do not resemble those of untreated tooth enamel. Nevertheless, both qualitative and quantitative surface evaluations clearly showed air-polishing with erythritol to be gentler on enamel than that with sodium bicarbonate.

### Clinical relevance

The average roughness of natural tooth enamel is indicated as approximately 1.5 µm [24]. Jones et al. observed that most of their study subjects could distinguish samples with a roughness between 0.06 µm and 3.43 µm with their tongues and correctly sort them according to their roughness [18]. Bollen et al. suggested that plaque accumulation increases with increased roughness of oral hard substances and only reaches a minimum at an average roughness of less than 0.2 µm [4]. The results of the current study show that the use of air-polishing devices can lead to a relevant increase in roughness on polished enamel samples, depending on the choice of prophy powder. While the surface changes caused by air-polishing with erythritol powder are rather negligible, a negative effect of periodical professional tooth cleaning with sodium bicarbonate powder regarding plaque accumulation cannot be ruled out completely at present. On the other hand, required treatment times with sodium bicarbonate were shown to be a quarter shorter compared to those with erythritol [30]. Therefore, the clinician has to make a trade-off between saving time and inadvertent removal of sound enamel.

## Conclusion

In the course of this study, an advanced scanning electron microscope (SEM)-based method for measuring submicroscopic roughness profiles of enamel samples was validated. The results of the study show that the regular use of sodium bicarbonate and erythritol powder during fixed appliance therapy lead to a highly significant increase in surface roughness. Furthermore, sodium bicarbonate was significantly more abrasive than erythritol, although powder-specific application times that match the clinical performance were considered.

## Supplementary Information

The supplementary material provides further information regarding the image processing and filtering and validation of the method. (Fiji macro, trueness and pricision data, figure of filtering diffrences)
