# Supplementary material for: Scanning electron microscopy evaluation of enamel surfaces using different air-polishing powders in the orthodontic setting: an in vitro study
Source: J Orofac Orthop. 2023 May 5;85(6):404–13. doi: 10.1007/s00056-023-00466-2 (PMC11496338; doi:10.1007/s00056-023-00466-2)
Supplement: Supplementary file 1 — The supplementary material provides further information regarding the image processing and filtering and validation of the method. (Fiji macro, trueness and pricision data, figure of filtering diffrences) [file 56_2023_466_MOESM1_ESM.pdf]

Supplementary Table 1 Online Resource 1: Fiji (imagej.net) macro for image processing and filtering

rename("1");
run("Multiply...", "value=1000000");
resetMinAndMax();
run("Set Scale...", "distance=1024 known=98.81 unit=um");
run("Duplicate...", " ");
run("Gaussian Blur...", "sigma=15 scaled");
rename("2");
imageCalculator("Subtract create 32-bit", "1","2");
close(1);
close(2);
//setTool("rectangle");
makeRectangle(200, 200, 624, 624);
run("Crop");

Supplementary Table 2 Online Resource 2: Validation – Test of precision for different roughness measurement protocols

	**S_a - Unfiltered**	**S_a - First order corrected**	**S_a - Filtered in Fiji**
Positioned top right	916.65 nm	226.83 nm	128.4 nm
Positioned top left	935.87 nm	220.05 nm	131.4 nm
Positioned bottom right	987.53 nm	232.15 nm	131.7 nm
Positioned bottom left	785.53 nm	232.07 nm	129.6 nm
Rotated 0°	928.02 nm	228.17 nm	127.5 nm
Rotated 90°	357.41 nm	224.11 nm	130.7 nm
Rotated 180°	885.37 nm	224.42 nm	128.2 nm
Rotated 270°	1480.00 nm	223.58 nm	127.4 nm
Working distance 4 mm	791.72 nm	246.86 nm	124.9 nm
Working distance 5.7 mm	928.02 nm	228.17 nm	127.5 nm
Working distance 7.2 mm	1170.00 nm	221.17 nm	131.4 nm
Working distance 8.7 mm	1210.00 nm	191.59 nm	125.3 nm
Working distance 10.2 mm	1210.00 nm	181.24 nm	125.8 nm
Overall Mean	971.50 nm	221.09 nm	128.525 nm
Overall standard deviation	269.19 nm	17.02 nm	2.34 nm
**Precision** (percentage deviation)	**27.71 %**	**7.70 %**	**1.82 %**

Fiji (imagej.net)

Supplementary Table 3 Online Resource 3: Validation **-** Test of trueness for different roughness measurement protocols

**Profile cut out**	**S_a - Unfiltered**	**S_a - First order corrected**	**S_a - Filtered in Fiji**
0-0.1 mm	1180 nm	54.21 nm	42.36 nm
0.1-0.2 mm	1240 nm	103.13 nm	27.64 nm
0.2-0.3 mm	1220 nm	73.40 nm	32.30 nm
0.3-0.4 mm	1150 nm	40.82 nm	30.16 nm
0.4-0.5 mm	1150 nm	52.67 nm	34.80 nm
0.5-0.6 mm	1190 nm	51.64 nm	22.75 nm
0.6-0.7 mm	1200 nm	52.86 nm	38.45 nm
0.7-0.8 mm	1240 nm	57.85 nm	30.61 nm
0.8-0.9 mm	1230 nm	95.72 nm	27.11 nm
0.9-1.0 mm	1260 nm	84.86 nm	34.35 nm
1.0-1.1 mm	1220 nm	70.05 nm	42.64 nm
1.1-1.2 mm	1270 nm	58.97 nm	45.22 nm
1.2-1.25 mm	1330 nm	65.72 nm	53.38 nm
Mean	1221.54 nm	66.30 nm	35.52 nm
**Trueness** (Difference to roughness normal)	**1148.04 nm**	**7.20 nm**	**37.98 nm**

Fiji (imagej.net)

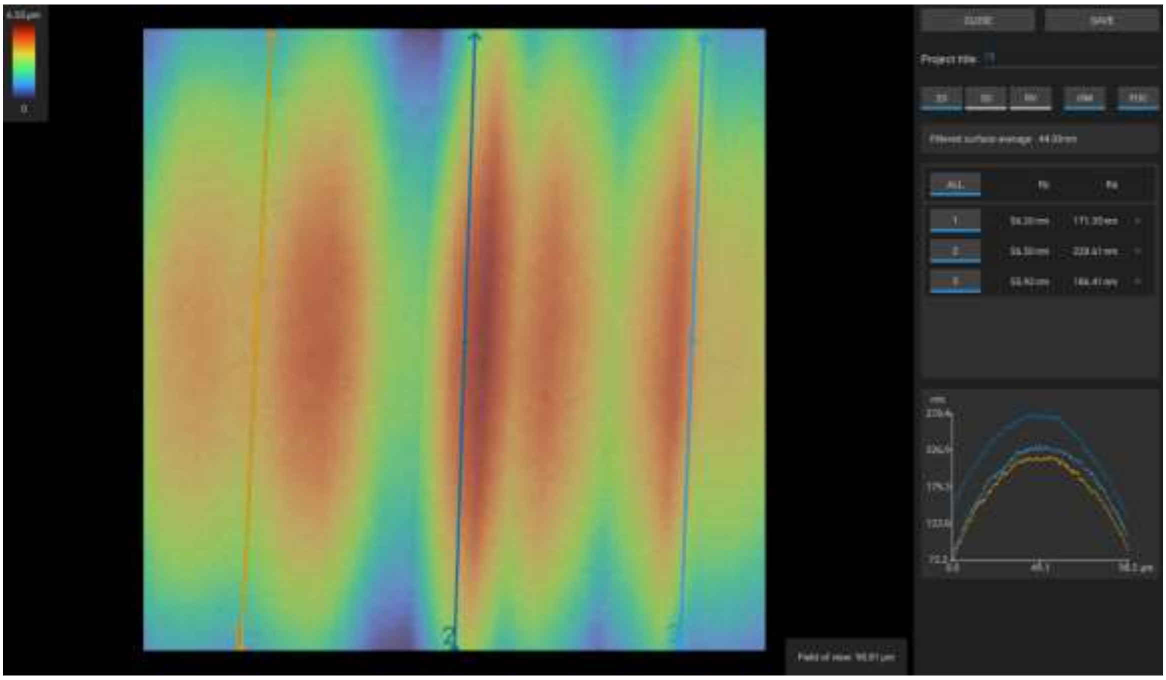

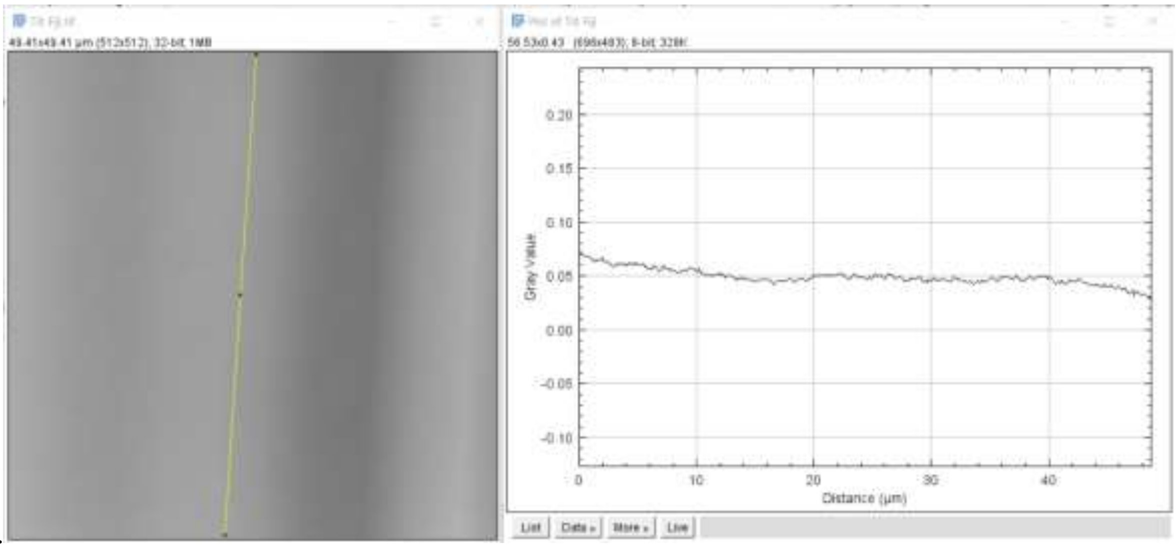

b.

Supplementary Figure 1 Online Resource 4: Surface of roughness normal after first order correction (a) and filtering in Fiji (imagej.net) (b)

(a) The roughness profile after first order correction with the 3D Roughness Reconstruction Software (Thermo Fisher Scientific, Hagen, Germany) is represented as a heatmap on the left side. Three exemplary line profiles (1, 2, 3) are plotted in the bottom right corner. One can notice an incomplete filtering of the curvature, which is the reason of higher roughness values in measurements of the trueness validation.

(b) The same profile after image processing and filtering in Fiji (imagej.net) in shown on the left side. The exemplary line profile shows a sufficient filtering when plotted as demonstrated on the right.
